# A systematic review and meta-analysis of effect of mutant traits in chickens

**DOI:** 10.1007/s11250-025-04624-z

**Published:** 2025-10-13

**Authors:** Salome Mhlabini, Thobela Louis Tyasi, Khetho Ratshilumela Nemutandani

**Affiliations:** Department of Agricultural Economics and Animal Production, University of Limpopo, Polokwane, South Africa

**Keywords:** Body weight, Feed intake, Egg weight, Egg number, Naked neck

## Abstract

Mutant traits in chickens, such as naked neck, frizzle, dwarfism, and featherless, have been associated with improved adaptability, productivity, and resilience under different environmental conditions. The study aimed to evaluate the effects of mutant traits on productivity outcomes in chickens through the systematic review and meta-analysis approach. A total of 164 articles were retrieved from Scholar, PubMed, ScienceDirect, and Web of Science databases and only 28 were used for systematic review while 12 were used for meta-analysis between the year 1993 and 2025, which contained information concerning 16,821 and 4939 chickens with mutant traits and without mutant traits, respectively. The pooled analysis revealed no significant effect of mutant traits on hen day egg production (2.86; 95% CI: −2.32; 8.03), egg number (0.43; 95% CI: −0.52; 1.38), egg weight (1.19; 95% CI: −0.95; 3.33), hatchability (−3.41; 95% CI: −7.78; −0.95), shell percentage (1.42; 95% CI: −0.28; 3.13), feed intake (1.65; 95% CI: −2.88; 6.18), body weight (11.29; 95% CI: −57.48; 80.05), mortality (0.63; 95% CI: −4.30, 5.56), feed conversion ratio (−0.06; 95% CI: −0.18, −0.06), and breast yield percentage (0.65; 95% CI: −0.08, 1.38). However, a significant positive effect was observed on dressing yield percentage (2.40; 95% CI: 1.05; 3.76) and giblet yield (0.42; 95% CI: 0.17, 0.68), while a significant reduction was noted in abdominal fat (−0.54; 95% CI: −1.00, −0.08). Overall, the results indicated high heterogeneity across the findings suggesting variability among the included studies. Mutant traits can enhance poultry traits, however their impact on productivity remains variable.

## Introduction

Phenotypic characteristics that arise from genetic mutations and exhibit distinct, discontinuous variations have been noted in chickens (Roulin 2004). These include Naked neck, Frizzle, Crest, Silky, Polydactyl, and Ptilopody which are caused by specific gene mutations affecting feather structure or limb development (Mensah 2017). These traits are controlled by major genes, and their expression can have implications for adaptability, productivity, and breed identity (Dorshorst et al. 2010; Adomako and Asamoah 2025). The most common include Naked neck, an incompletely dominant mutation associated with reduced feather coverage on the neck and improved thermotolerance (Desta 2021); Frizzle, an autosomal incompletely dominant mutation in chickens that affects feather structure, chickens with this trait exhibit feathers that curl outward and upward (Dong et al. 2018); Crest, an incompletely dominant mutation resulting in a feather tuft on the head (Li et al. 2021); Silky, a recessive mutation in PMEL17 that causes a silky feather texture (Ng and Li 2018); Polydactyl, a dominant mutation leading to the presence of extra toes (Bubshait 2022); Ptilopody, an autosomal dominant mutation resulting in feathered shanks and toes (Bortoluzzi et al. 2020). Various studies have pointed out that the economic benefits of mutant traits in the current chicken breeding system originate from their strong disease resistance, average reproductive performance, and excellent tolerance to tropical climates (Magothe et al. 2010; Mensah et al. 2023). Nonetheless, the impact of these mutant traits on chicken productivity has not yet been fully explored. Studies have been conducted to evaluate the effectiveness of these mutant traits in enhancing the productivity of the chickens (Nwachukwu and Nwabuko 2012; Faruque et al. 2018). Recent advances in genetics have shown a number of chicken mutant traits that could provide novel methods to increase production (Magothe et al. 2010; Ahmed et al. 2012; Faruque et al. 2018). However, the findings of the recent studies on the effect of mutant traits on chickens’ production differs. With Naked neck often improved hen-day egg production (Nosike et al. 2017; Faruque et al. 2018; Oleforuh-Okoleh et al. 2021; Alam et al. 2022) and body weight (Haque and Howlider 2000; Ige et al. 2012; Talukder et al. 2016; Faruque et al. 2018; Alam et al. 2022), though effects on clutch size, total egg number, feed intake, and egg weight were inconsistent (Sa et al. 2006; Ahmed et al. 2012; Talukder et al. 2016; Mensah et al. 2023). Frizzle traits generally increased body weight (Magothe et al. 2010; Ige et al. 2012), with limited effects on egg production and egg quality (Subalini et al. 2014; Mensah et al. 2023). Polydactyl chickens showed significant improvements in egg number and hen-day production (Mensah et al. 2023). Crest traits had minimal impact on egg production or hatchability (Magothe et al. 2010; Mensah et al. 2023). Silky traits affected hatchability in some studies but had little influence on egg production (Subalini et al. 2014; Mensah et al. 2023). Hence, it is important to conduct systematic review and meta-analysis to resolve the contrast. The knowledge on the systematic review and meta-analysis on the effect of mutant traits in chickens is limited. Thus, this study aimed to comprehensively review and meta-analyse the effect of mutant traits in chickens. By evaluating the findings, this review seeks to provide information on the potential benefits and limitations of introducing chickens with mutant traits and to inform the development of evidence-based breeding strategies for the poultry industry.

## Materials and methods

### Eligibility criteria

In this review, the research question was framed using the Population–Intervention–Comparison–Outcome (PICO) approach. The population comprised chickens of diverse breeds and genetic backgrounds, with the intervention being the presence of specific mutant traits (Naked Neck, Frizzle, Crest, Silky, Polydactyl, and Ptilopody). These were compared with normal-feathered or standard phenotypes, and the outcomes assessed included growth performance (body weight, feed intake, feed conversion ratio), reproductive traits (hen-day egg production, egg number, egg weight, hatchability, shell percentage), carcass characteristics (dressing yield, breast yield, giblet yield, abdominal fat), and selected egg quality parameters. A preliminary search of the PICO components on PubMed, Google Scholar, Web of Science, and ScienceDirect databases was conducted before deciding to carry out the study.

### Search strategy

The search of published studies was conducted from the database of Google Scholar, PubMed, ScienceDirect, and Web of Science. The following keywords: “mutant traits/silkie/polydactyl/ptilopody/naked neck/frizzle/crest”, and “chicken” were searched.

### Inclusion criteria

The titles and abstracts were manually screened to identify articles that investigated the effects of mutant traits in chickens. Articles meeting this criterion were included in this review. Articles were included given that they were written in English, evaluating one or more of the following traits—Naked Neck, Frizzle, Crest, Silky, Polydactyl, or Ptilopody; any breeds, strains, or crossbreds of chickens; experimental, observational, or on-farm trials; they reported on at least one relevant productivity outcome.

### Exclusion criteria

The exclusion criteria included articles without the keyword combination, duplicates, studied other species other than chicken, or did not investigate the effects of the mutant traits in chickens, absence of quantitative data for meta-analysis, reviews, unpublished articles, and conference abstracts.

### Data extraction

The data was extracted from all the studies that met the inclusion criteria, author, year of publication and the type of mutant trait studied.

### Assessment of risk of bias

Joanna Briggs Institute (JBI) tool (Aromataris et al. 2015) was used to assess the quality of the articles selected in this study. The checklist consists of six questions, namely:

1. Were the criteria for inclusion in the sample clearly defined?
2. Were the study subjects and the setting described in detail?
3. Was the exposure measured in a valid and reliable way?
4. Were the standard criteria used for measurement of the condition?
5. Were the outcomes measured in a valid and reliable way?
6. Was appropriate statistical analysis used?

Individual questions were examined against each of the selected articles and answers was given as ‘Yes’ and ‘No’.

### Statistical analysis

R software version 4.3.3 (The R Foundation for Statistical Computing) using the meta package was used for analysis. The “metafor” package was used to generate the meta-analysis statistics and forest plots (Viechtbauer 2010). The effects of different mutant traits on chicken performance were examined using a random effects model. The I^2^ statistics were used to test heterogeneity among studies.

### Test for publication bias

Publication bias refers to the tendency for studies with statistically significant results to be published more often than studies with non-significant findings, potentially overestimating effect sizes (Sutton et al. 2000). We intended to assess publication bias using funnel plot symmetry and Egger’s regression test when data conditions were met (≥10 trials and I^2^ < 50%) (Ioannidis and Trikalinos 2007). Heterogeneity, quantified using the I^2^ statistic, represents the proportion of variability in effect estimates due to differences between studies rather than sampling error (Higgins and Thompson 2002). Due to the high heterogeneity (I^2^ > 50%) and limited number of trials, statistical assessment of publication bias was not feasible.

### Ethical considerations

As a systematic review and meta-analysis, no new data were generated from human participants or animals, and therefore formal ethical approval was not required. However, several ethical issues were considered throughout the process. First, we ensured responsible use of existing data by accurately reporting study findings and avoiding selective presentation or misrepresentation. Second, we maintained academic integrity by citing all included studies appropriately and avoiding plagiarism, duplicate publication, or data fabrication. Third, transparency and reproducibility were emphasized by providing detailed descriptions of the search strategy, inclusion and exclusion criteria, and data extraction process.

## Results

### Searched results

**Fig. 1:**
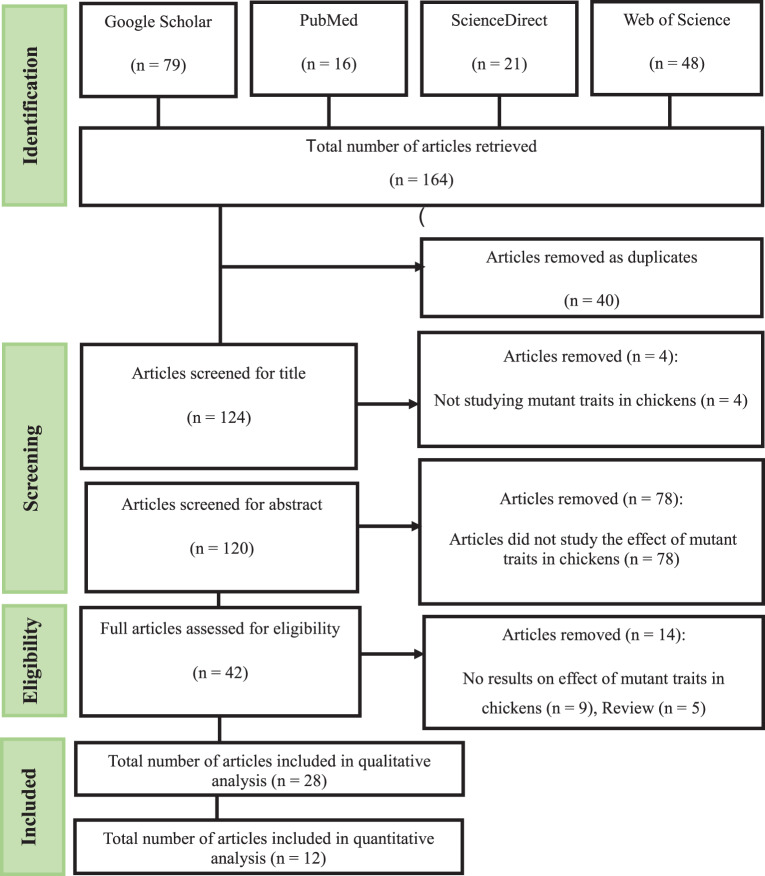
Flow chart of identification and selection of studies used in the systematic review and meta-analysis

Figure 1 shows the flowchart of the identification and selection of studies for the systematic review. In the primary search, a total of hundred and sixty-one articles (*n* = 164) were retrieved. Out of the hundred and sixty-one (*n* = 164) articles, forty (*n* = 40) were removed as duplicates, eighty-two (*n* = 82) were removed when screening for title and abstract. A total of fourteen articles (*n* = 14) were removed during the full-text search and eligibility verification. A total of twenty-eight articles (*n* = 28) were included in the systematic review and twelve (*n* = 12) were included in meta-analysis.

### Characteristics of included studies

Table 1 shows the characterization of the included articles. The results indicated that twenty-six articles (*n* = 26) studied naked neck as a mutant trait. Silky, ptilopody, polydactyl were the least (*n* = 1) studied mutant traits (Mensah et al. 2023). Five articles (*n* = 5) were done in Bangladesh (Haque and Howlider 2000; Ahmed et al. 2012; Talukder et al. 2016; Faruque et al. 2018; Alam et al. 2022).

**Table 1:** Characteristics of included studies

Author	Year	Country	Mutant traits
Ahmed et al.	2012	Bangladesh	Naked neck
Alam et al.	2022	Bangladesh	Naked neck
Amad and Liebert	2016	Germany	Naked neck
Asumah et al.	2022	Ghana	Naked neck, frizzle feather, Naked neck- Frizzle
Azoulay et al.	2011	Israel	Featherless
Cahaner et al.	2008	Israel	Naked neck, featherless
Dunga et al.	2025	Ghana	Naked neck, frizzle feather
Ebehart and Washburn	1993	Georgia	Naked neck
Faruque et al.	2018	Bangladesh	Naked neck
Gala et al.	2010	Egypt	Naked neck
Hadad et al.	2014	Israel	Featherless
Haque and Howlider	2000	Bangladesh	Naked neck
Ige et al.	2012	Nigeria	Frizzle feathered, naked neck
Magothe et al.	2010	Kenya	Crest, frizzle, naked neck
Mensah et al.	2023	Ghana	Silky, frizzle, naked neck, crest, polydactyl, ptilopody
Nosike et al.	2017	Nigeria	Naked neck, frizzle feather
Nwachukwu et al.	2012	Nigeria	Naked neck, frizzle feather
Oleforuh-Okolen et al.	2021	Nigeria	Naked neck
Pavlovski et al.	2009	Republic of Serbia	Naked neck
Rajkumar et al.	2011	India	Naked neck
SA et al.	2006	Egypt	Naked neck
Shafig et al.	2021	Pakistan	Naked neck
Shafiq et al.	2023	Pakistan	Naked neck
Subalini et al.	2014	Sri Lanka	Frizzle, Naked neck-Frizzle
Talukder	2016	Bangladesh	Naked neck
Tóth et al.	2021	Hungary	Naked neck
Usman et al.	2014	Pakistan	Naked neck
Yunis and Cahaner	1999	Israel	Naked neck, frizzle feather

### Publication by year

**Fig. 2:**
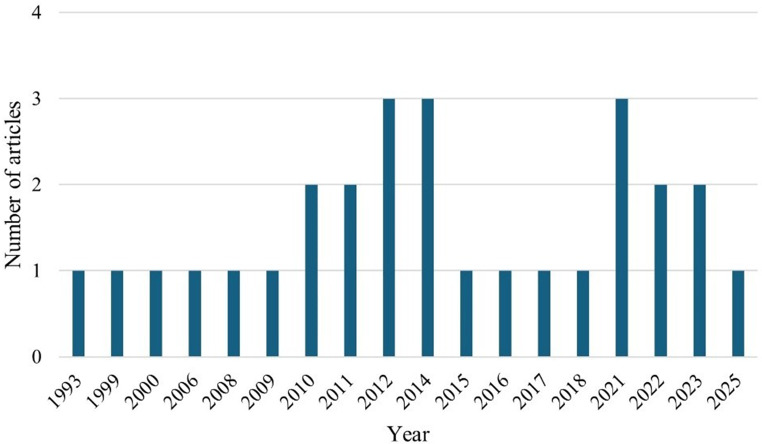
Publication by year

Figure 2 shows the publication of articles by year. The results showed that three articles (*n* = 3) were published in the years 2012, 2014, and 2021. The years 1993 (Eberhart and Washburn 1993), 1999 (Yunis and Cahaner 1999), 2000 (Haque and Howlider 2000), 2006 (Sa et al. 2006), 2008 (Cahaner et al. 2008), 2009 (Pavlovski et al. 2009), 2015 (Amad and Liebert 2016), 2016 (Talukder et al. 2016), 2017 (Nosike et al. 2017), 2018 (Faruque et al. 2018), 2025 (Dunga et al. 2025) had one published article respectively.

### Publication by country

**Fig. 3:**
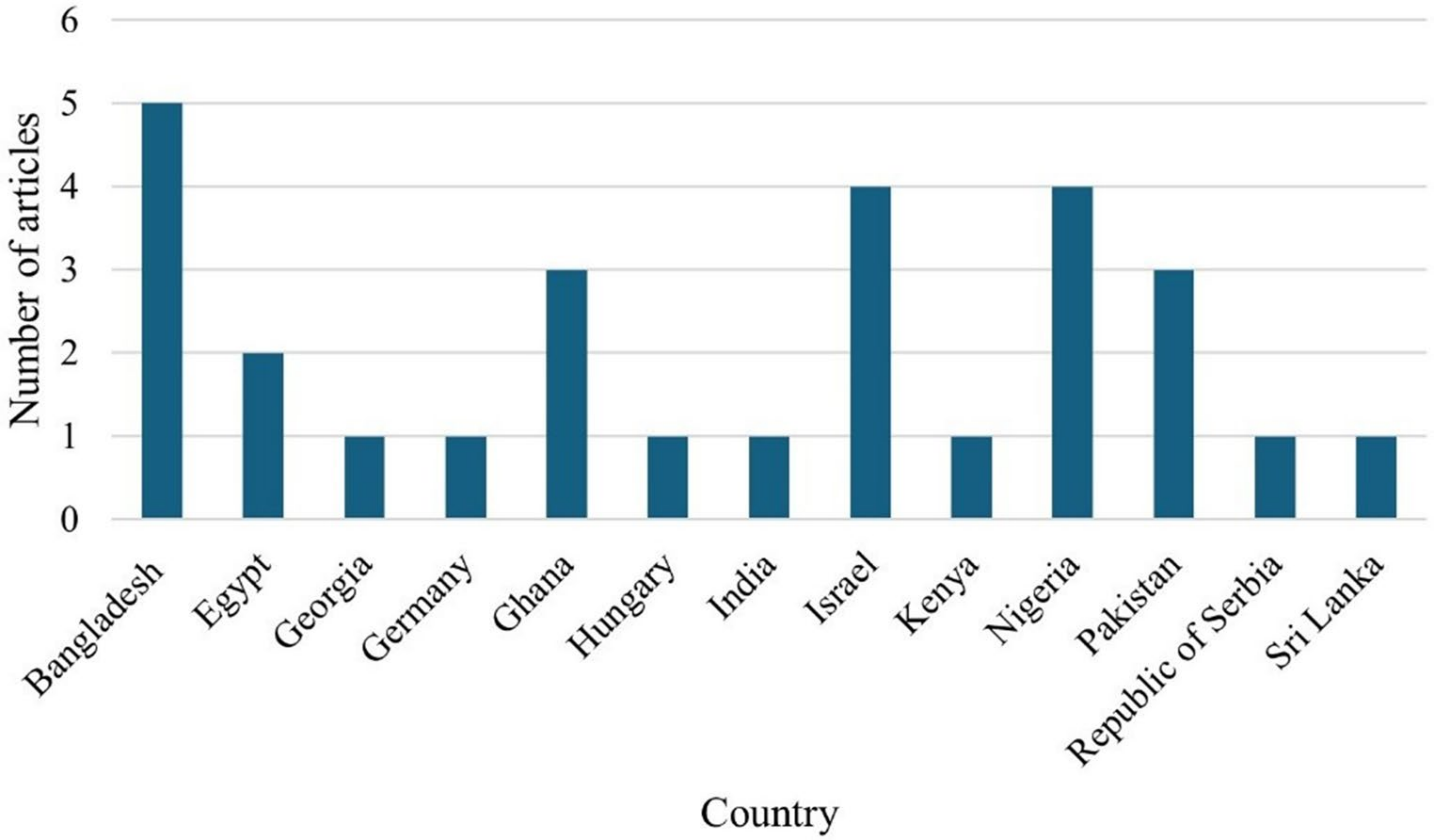
Publication by country

Figure 3 shows the publication of articles by country. The results indicated that five articles (*n* = 5) were from Bangladesh (Haque and Howlider 2000; Ahmed et al. 2012; Talukder et al. 2016; Faruque et al. 2018; Alam et al. 2022). The following countries: Georgia (Eberhart and Washburn 1993), Republic of Serbia (Pavlovski et al. 2009), Kenya (Magothe et al. 2010), India (Rajkumar et al. 2011), Germany (Amad and Liebert 2016), and Hungary (Tóth et al. 2021) published one article (*n* = 1) each.

### Distribution of articles by journal

**Fig. 4:**
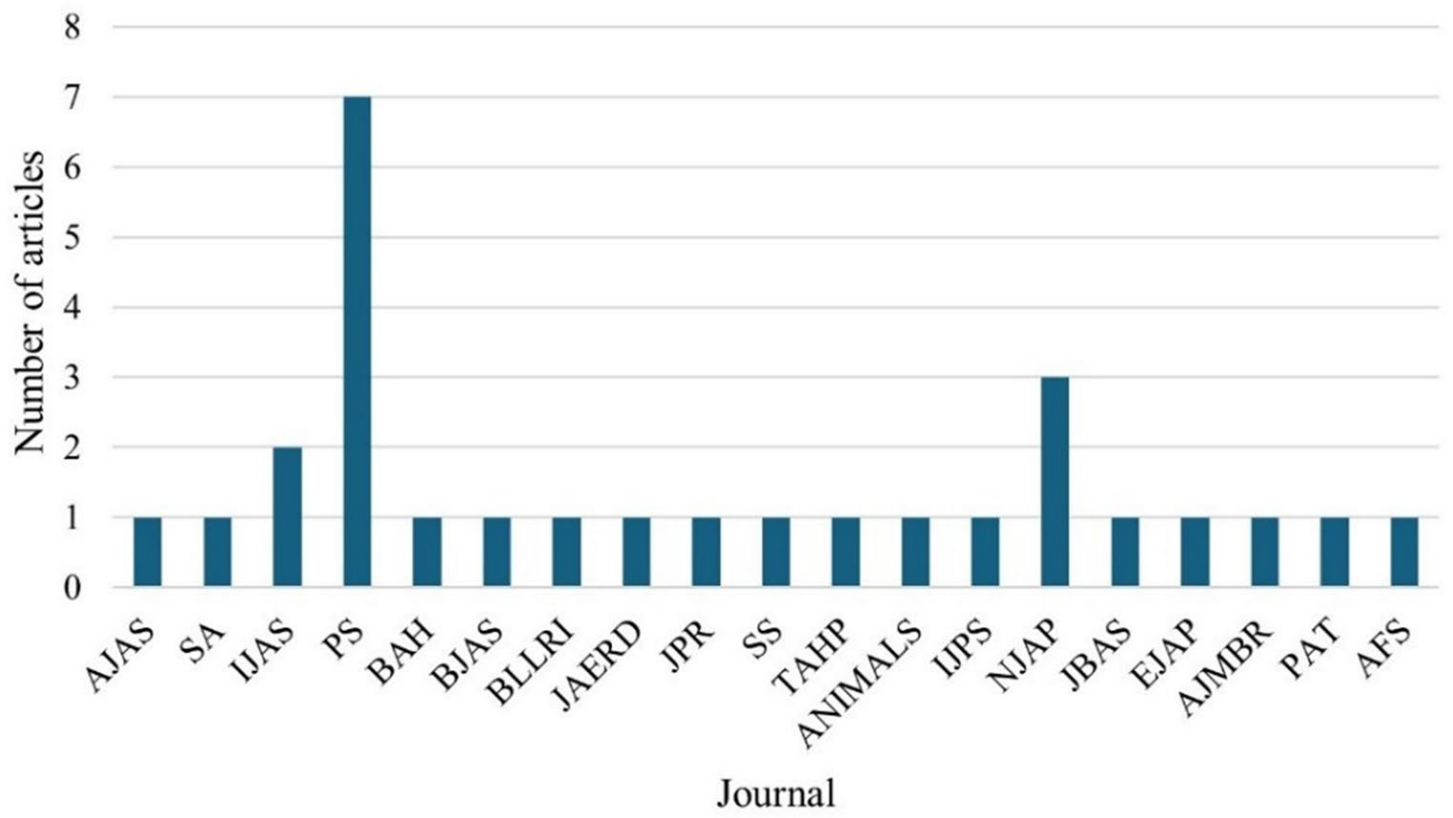
Distribution of articles by journal. IJPS = International Journal of Poultry Science, NJAP = Nigerian Journal of Animal Production, JBAS = Journal of Basic and Applied Science, PS = Poultry Science, EJAP = Egyptian Journal of Animal Production, AJMBR = Asian Journal of Medical and Biological Sciences, AFS = Agricultural and Food Sciences, AJAS = Asian-Australian Journal of Animal Sciences, SA = Scientific African, IJAS = Indian Journal of Animal Sciences, BAH = Biodiversity in Animal Husbandry, BJAS = Bangladesh Journal of Animal Science, BLRI = Bangladesh Livestock Research Institute, JAERD = Journal of Agricultural Extension and Rural Development, JPR = Journal of Poultry Research, SS = Springer Science, TAHP = Tropical Animal Health and Production

Figure 4 presents the distribution of articles by journal. The results showed that seven articles (*n* = 7) got published by Poultry science, three articles (*n* = 3) were published under the Nigerian Journal of Animal Production (Nwachukwu and Nwabuko 2012; Nosike et al. 2017; Oleforuh-Okoleh et al. 2021).

### Distribution of articles by mutant traits

**Fig. 5:**
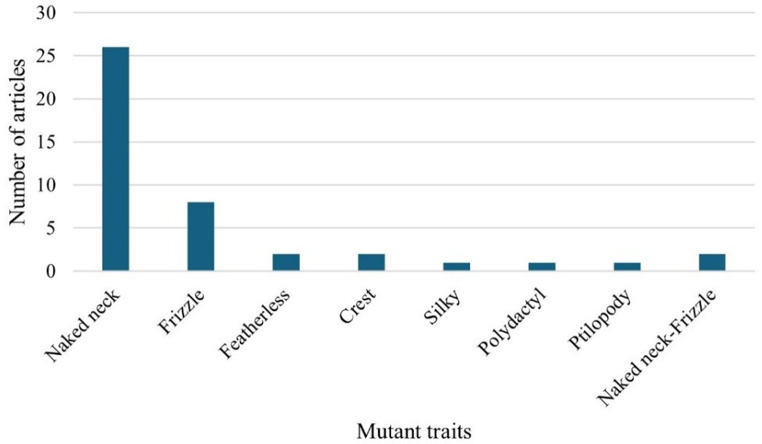
Distribution of articles by mutant traits

Figure 5 shows the distribution of articles by mutant traits. The results showed that the neck naked trait was the most studied mutant trait (*n* = 26), while silky (Mensah et al. 2023), polydactyl (Mensah et al. 2023), and ptilopody (Mensah et al. 2023) were studied in one article (*n* = 1) each.

### Effect of mutant traits on hen day egg production

**Fig. 6:**
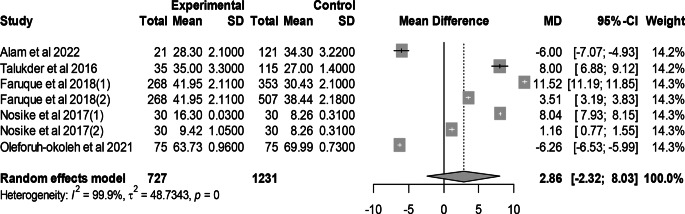
Effects of mutant traits on hen day egg production: forest plot

Meta-analysis of 7 trials from 5 articles demonstrated no significant effect of mutant traits on hen day egg production compared with normal chickens as control (MD = 2.86, 95% CI: −2.32, 8.03; Fig. 6). Heterogeneity was evident among the 7 trials (I^2^ = 99.9%).

### Effect of mutant traits on egg number

**Fig. 7:**
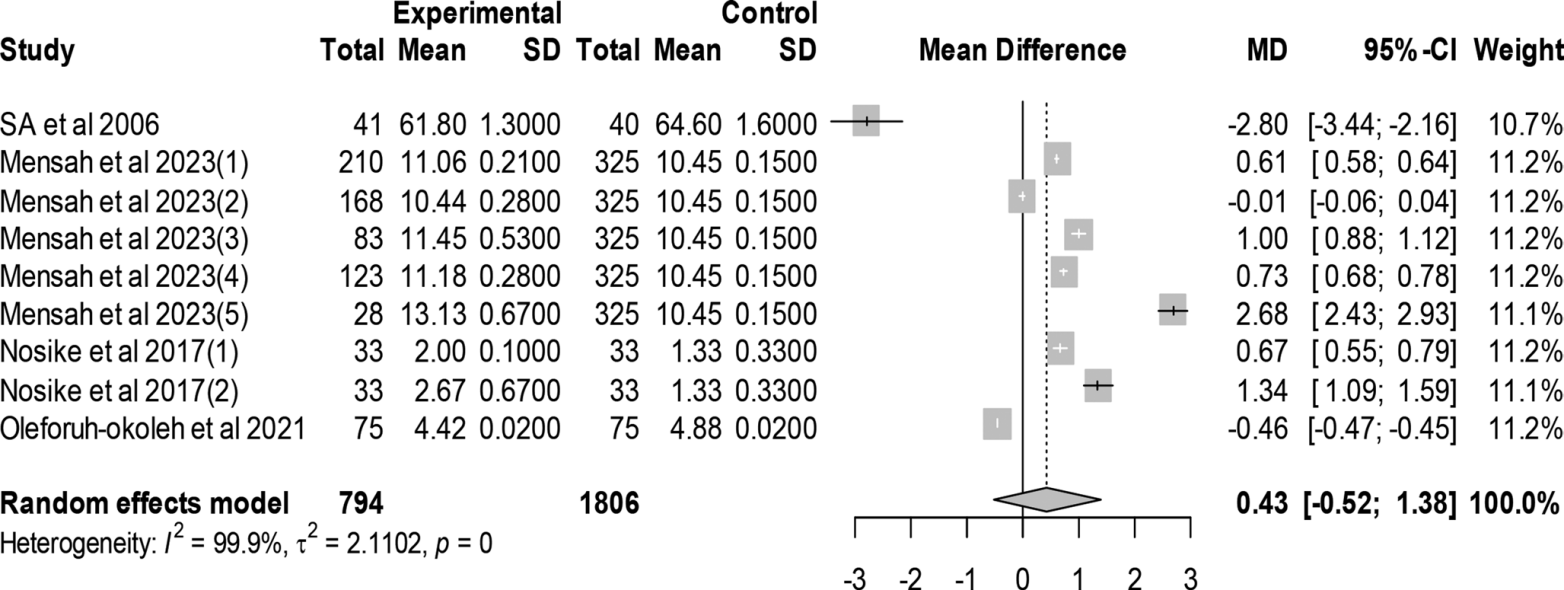
Effects of mutant traits on egg number: forest plot

Meta-analysis of 9 trials from 4 articles revealed no significant effect of mutant traits on egg number as compared with normal chickens as control (MD = 0.43, 95% CI: −0.52, 1.38; Fig. 7). Heterogeneity was evident among the 9 trials (I^2^ = 99.9%).

### Effect of mutant traits on egg weight

**Fig. 8:**
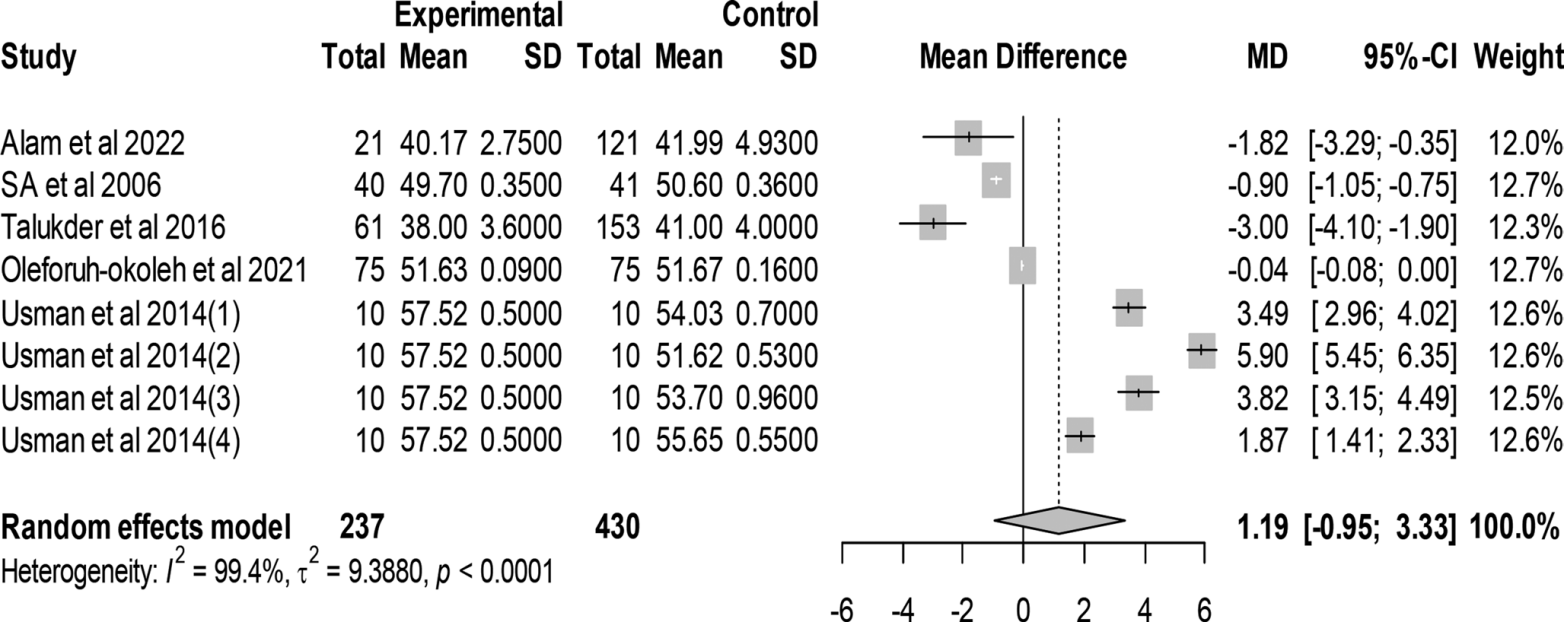
Effects of mutant traits on egg weight: forest plot

Meta-analysis of 8 trials from 5 articles revealed no significant effect of mutant traits on egg weight as compared with normal chickens as control (MD = 1.19, 95% CI: −0.95, 3.33; Fig. 8). Heterogeneity was evident among the 8 trials (I^2^ = 99.4%).

### Effect of mutant traits on hatchability

**Fig. 9:**
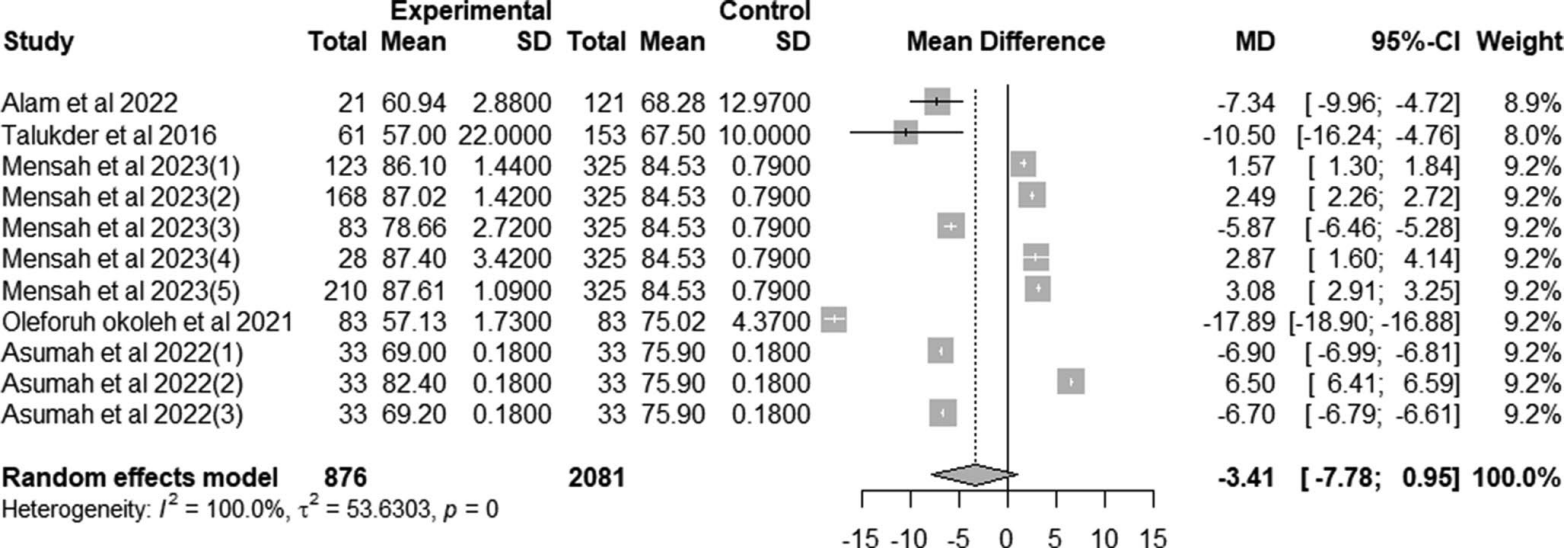
Effects of mutant traits on hatchability: forest plot

Meta-analysis of 11 trials from 5 articles revealed no significant effect of mutant traits on hatchability as compared with normal chickens as control (MD = −3.41, 95% CI: −7.78, 0.95; Fig. 9). Heterogeneity was evident among the 11 trials (I^2^ = 100%).

### Effect of mutant traits shell percentage

**Fig. 10:**
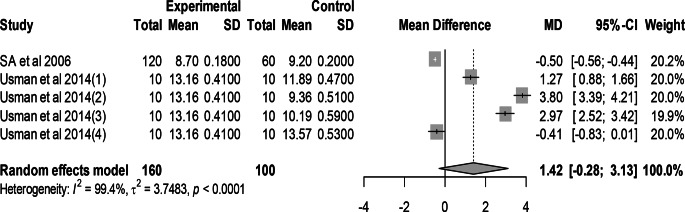
Effects of mutant traits on Shell percentage: forest plot

Meta-analysis of 5 trials from 2 articles revealed no significant effect of mutant traits on shell percentage as compared with normal chickens as control (MD = 1.42, 95% CI: −0.28, 3.13; Fig. 10). Heterogeneity was evident among the 5 trials (I^2^ = 99.4%).

### Effect of mutant traits on feed intake

**Fig. 11:**
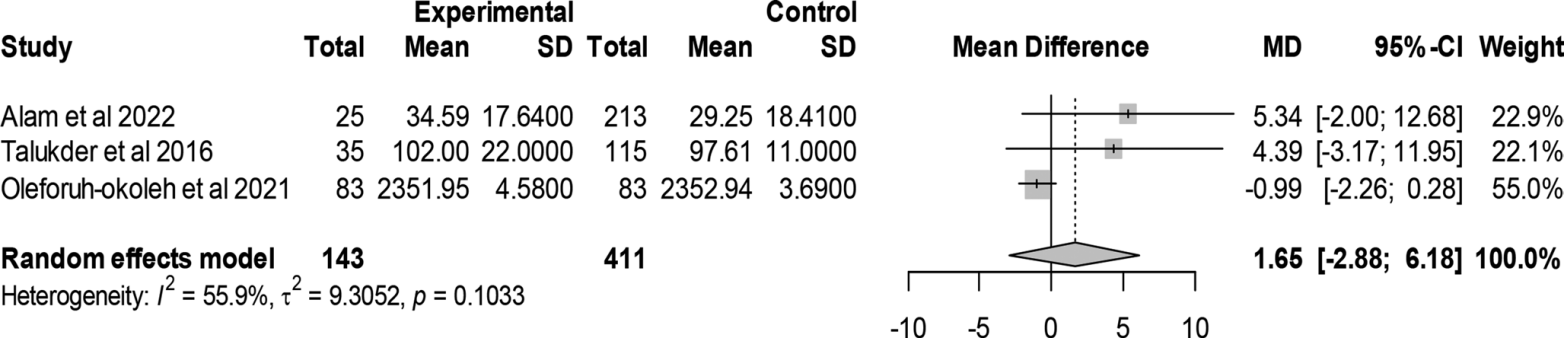
Effects of mutant traits on feed intake: forest plot

Meta-analysis of 3 trials from 3 articles revealed no significant effect of mutant traits on feed intake as compared with normal chickens as control (MD = 1.65, 95% CI: −2.88, 6.18; Fig. 11). Heterogeneity was evident among the 3 trials (I^2^ = 55.9%).

### Effect of mutant traits on body weight

**Fig. 12:**
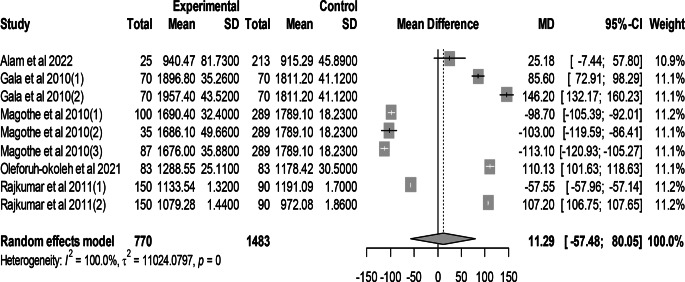
Effects of mutant traits on body weight: forest plot

Meta-analysis of 9 trials from 5 articles revealed no significant effect of mutant traits on body weight as compared with normal chickens as control (MD = 11.29, 95% CI: −57.48, 80.05; Fig. 12). Heterogeneity was evident among the 9 trials (I^2^ = 100.00%).

### Effect of mutant traits on mortality

Meta-analysis of 3 trials from 3 articles revealed no significant effect of mutant traits on mortality as compared with normal chickens as control (MD = 0.63, 95% CI: −4.30, 5.56; Fig. 1^3^). Heterogeneity was evident among the 3 trials (I^2^ = 98.2%).

**Fig. 13:**
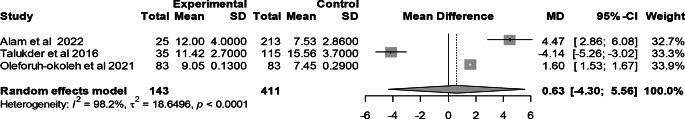
Effects of mutant traits on mortality: forest plot

### Effect of mutant traits on feed conversion ratio

**Fig. 14:**
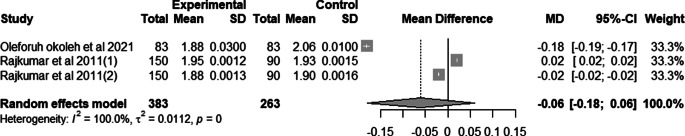
Effects of mutant traits on feed conversion ratio: forest plot

Meta-analysis of 3 trials from 2 articles revealed no significant effect of mutant traits on feed conversion ratio as compared with normal chickens as control (MD = −0.06, 95% CI: −0.18, 0.06; Fig. 14). Heterogeneity was evident among the 3 trials (I^2^ = 100.00%).

### Effect of mutant traits on dressing yield percentage

**Fig. 15:**
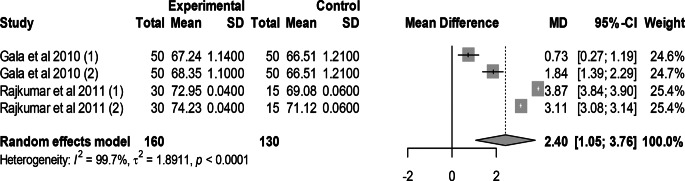
Effects of mutant traits on dressing: forest plot

Meta-analysis of 4 trials from 2 articles revealed no significant effect of mutant traits on dressing yield percentage as compared with normal chickens as control (MD = 2.40, 95% CI: 1.05, 3.76; Fig. 15). Heterogeneity was evident among the 4 trials (I^2^ = 99.7%).

### Effect of mutant traits on giblets

**Fig. 16:**
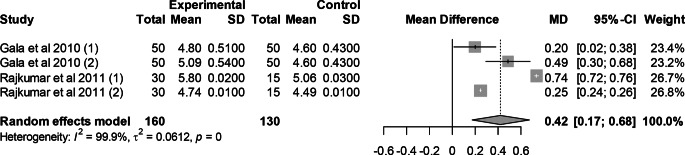
Effects of mutant traits on giblets: forest plot

Meta-analysis of 4 trials from 2 articles revealed no significant effect of mutant traits on giblets as compared with normal chickens as control (MD = 0.42, 95% CI: 0.17, 0.68; Fig. 16). Heterogeneity was evident among the 4 trials (I^2^ = 99.9%).

### Effect of mutant traits on breast yield percentage

**Fig. 17:**
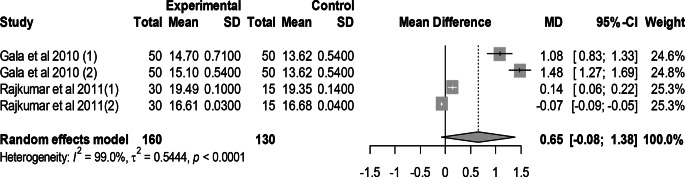
Effects of mutant traits on breast yield percentage: forest plot

Meta-analysis of 4 trials from 2 articles revealed no significant effect of mutant traits on breast yield percentage as compared with normal chickens as control (MD = 0.65, 95% CI: −0.08, 1.38; Fig. 17). Heterogeneity was evident among the 4 trials (I^2^ = 99.0%).

### Effect of mutant traits on abdominal fat

**Fig. 18:**
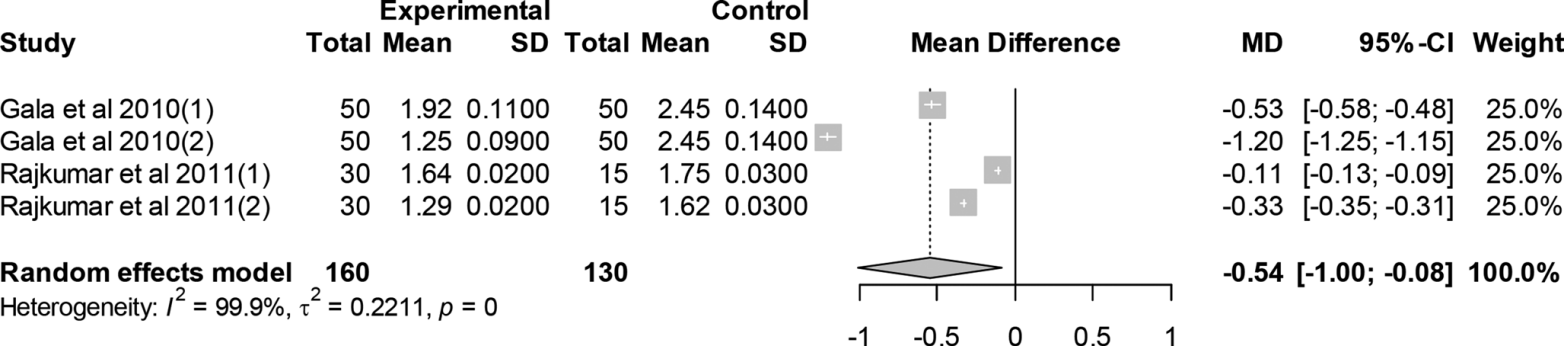
Effects of mutant traits on abdominal fat: forest plot

Meta-analysis of 4 trials from 2 articles revealed significant effect of mutant traits on abdominal fat as compared with normal chickens as control (MD = −0.54, 95% CI: −1.00, −0.08; Fig. 18). Heterogeneity was evident among the 4 trials (I^2^ = 99.9%).

## Discussion

Mutant traits such as, naked neck and frizzle feathering have been linked to improved heat tolerance and feed conversion efficiency, making them important in breeding programs aimed at enhancing poultry performance in different environments (Sa et al. 2006; Rajkumar et al. 2011). This review aimed to evaluate the existing evidence on mutant traits in chickens and their effects on productivity outcomes using the systematic review and meta-analysis approach. A total of 28 articles were included in this systematic review, with 12 been used for meta-analysis published from the year 1993 and 2025 involving 21,760 chickens. Out of 28 articles, 45% investigated the effects of mutant traits on egg production. The pooled meta-analysis revealed that mutant traits had an influence on abdominal fat (Gala et al. 2010; Rajkumar et al. 2011). Abdominal fat is an economically important trait in broilers, while excessive abdominal fat is often considered undesirable, as it reduces carcass yield and consumer acceptance, although it may reflect energy reserves (Gala et al. 2010; Rajkumar et al. 2011). There was no evidence of mutant traits influencing the hen-day egg production (Talukder et al. 2016; Nosike et al. 2017; Faruque et al. 2018; Oleforuh-Okoleh et al. 2021; Alam et al. 2022), egg number (Sa et al. 2006; Nosike et al. 2017; Oleforuh-Okoleh et al. 2021; Mensah et al. 2023), egg weight (Sa et al. 2006; Usman et al. 2014; Talukder et al. 2016; Oleforuh-Okoleh et al. 2021; Alam et al. 2022), hatchability (Talukder et al. 2016; Oleforuh-Okoleh et al. 2021, Alam et al. 2022; Mensah et al. 2023), shell percentage (Sa et al. 2006; Usman et al. 2014), feed intake (Talukder et al. 2016; Oleforuh-Okoleh et al. 2021; Alam et al. 2022), mortality (Talukder et al. 2016; Oleforuh-Okoleh et al. 2021; Alam et al. 2022), feed conversion ratio (Rajkumar et al. 2011; Oleforuh-Okoleh et al. 2021), dressing yield percentage (Gala et al. 2010; Rajkumar et al. 2011), giblets (Gala et al. 2010; Rajkumar et al. 2011), breast meat (Gala et al. 2010; Rajkumar et al. 2011), though substantial variability was observed across trails. Gala et al. (2010) and Rajkumar et al. (2011) stated that body weight is a primary selection criterion in broiler breeding, as it directly affects growth performance. In layers, traits such as hen-day egg production and hatchability are critical for reproductive efficiency and profitability. Hen-day egg production provides insight into the consistency and productivity of laying hens (Talukder et al. 2016; Nosike et al. 2017), while hatchability is a key determinant of reproductive success and flock sustainability (Alam et al. 2022; Mensah et al. 2023). The findings of this meta-analysis suggest that mutant traits, such as Naked neck, crest, and frizzle, while having some influence on poultry characteristics such as body weight and abdominal fat, do not show consistent or significant effects across other parameters such as egg production or hatchability. The findings suggest that mutant traits may be useful for reducing abdominal fat in broilers, which could improve carcass quality and production efficiency. However, the lack of association between mutant traits and reproductive traits such as hen-day egg production and hatchability indicate these traits may be more influenced by environmental factors or complex genetics. The variability across studies further emphasizes the importance of considering genotype-by-environment interactions in breeding strategies. A key strength of this review is that no similar comprehensive systematic review and meta-analysis has been conducted on the effects of mutant traits across multiple performance parameters in poultry. This study contributes a broader understanding of the influence of genetic mutations on poultry productivity, integrating a wide range of studies and outcomes that were previously analysed individually. This study fills an important gap in the existing literature by synthesizing results from various trails on mutant traits and their effects on poultry performance. It provides valuable insights into the complex relationships between genetic mutations and various poultry characteristics, offering a foundation for future genetic studies and breeding strategies aimed at enhancing productivity. Several limitations were identified in the study, including small sample sizes (*n* < 10) in individual trials, which hindered the assessment of publication bias and moderator effects. High heterogeneity across studies made it difficult to draw firm conclusions about mutant trait effects. Additionally, differences in breeds and environmental conditions introduced variability, and the limited number of studies on certain traits reduced the overall reliability and generalizability of the findings.

In conclusion, while mutant traits show some promise in influencing certain poultry characteristics such as body weight and abdominal fat, their impact on other performance parameters such as egg production and hatchability remains inconsistent. This study emphasizes the need for more researches to fully understand the potential of mutant traits in poultry breeding. Despite the variability in findings, the study lays the groundwork for further exploration into how genetic mutations can be leveraged for improved poultry productivity.

## Data Availability

Data available upon request.
